# Training needs assessment: tool utilization and global impact

**DOI:** 10.1186/s12909-021-02748-y

**Published:** 2021-05-31

**Authors:** Adelais Markaki, Shreya Malhotra, Rebecca Billings, Lisa Theus

**Affiliations:** 1 PAHO/WHO Collaborating Center for International Nursing, Birmingham, AL USA; 2 School of Nursing, University of Alabama at Birmingham, 1701 University Boulevard, Birmingham, AL 35294 USA; 3 Stanford University, Stanford, CA USA

**Keywords:** Training needs assessment, Instrument, Psychometrics, Hennessy-Hicks, Capacity building, Continuous professional development, Interprofessional, Quality improvement, Healthcare organization

## Abstract

**Background:**

Global demand for standardized assessment of training needs and evaluation of professional continuing education programs across the healthcare workforce has led to various instrumentation efforts. The Hennessy-Hicks Training Needs Analysis (TNA) questionnaire is one of the most widely used validated tools. Endorsed by the World Health Organization, the tool informs the creation of tailored training to meet professional development needs. The purpose of this project was to describe TNA tool utilization across the globe and critically appraise the evidence of its impact in continuous professional development across disciplines and settings.

**Methods:**

A systematic integrative literature review of the state of the evidence across PubMed, Scopus, CINAHL, and Google Scholar databases was carried out. Full-text, peer reviewed articles and published dissertations/theses in English language that utilized the original, adapted or translated version of the TNA tool were included. Selected articles were appraised for type and level of evidence.

**Results:**

A total of 33 articles were synthesized using an inductive thematic approach, which revealed three overarching themes: individual, team/interprofessional, and organizational level training needs. Included articles represented 18 countries, with more than two thirds involving high-income countries, and one third middle-income countries. Four studies (12.1%) used the original English version instrument, 23 (69.7%) adapted the original version, and 6 (18.2%) translated and culturally adapted the tool. Twenty-three studies targeted needs at the individual level and utilized TNA to determine job roles and responsibilities. Thirteen articles represented the team/interprofessional theme, applying the TNA tool to compare training needs and perceptions among professional groups. Last, three articles used the tool to monitor the quality of care across an institution or healthcare system, demonstrating the organizational training needs theme.

**Conclusions:**

Overall evidence shows that the TNA survey is widely used as a clinical practice and educational quality improvement tool across continents. Translation, cultural adaptation, and psychometric testing within a variety of settings, populations, and countries consistently reveals training gaps and outcomes of targeted continuous professional development. Furthermore, it facilitates prioritization and allocation of limited educational resources based on the identified training needs. The TNA tool effectively addresses the “know-do” gap in global human resources for health by translating knowledge into action.

## Background

Over the last 25 years, a trained workforce has been at the core of success for any organization or industry. Appropriate and systematic approaches to training have been shown to result in skills improvement, which in turn raise the quality of employees [1]. Assessing and understanding workforce training needs ensures confidence, know-how, and a variety of new skills that bolster preparedness on an individual and team-based level in any organization [2]. Given the rapid technological advances, persisting workforce shortages, increased disease burden, and shrinking resources, healthcare organizations must methodically survey existing and expected performance levels of their staff [3]. Yet, evaluation of training and development processes, used until the mid-90’s, showed that healthcare professionals were not acquiring the necessary skills to successfully perform their jobs [4]. Similarly, healthcare organizations often did not carry out adequate assessments of training needs, due to limited time and resources, or failure to use research evidence to inform practice [5]. Consequently, training needs analysis (TNA) must be viewed and carried out in the context of existing healthcare systems to be consistent with the needs of employees and relevant to the ever-changing demands of organizations [6].

Literature on healthcare employee training needs has evolved considerably. Several TNA models were developed to understand and address training deficiencies in the workplace through data collection and analysis from both employees and employers [7]. The *traditional model* focuses on job behavior and task analysis, using surveys and formal interviews to gather data [8]. Its main drawback, besides being time-consuming, is its focus on predetermined outcomes which precludes the possibility of unplanned learning taking place. As an alternative, the *practical model* considers a trainer-centered, demand-led or supply-led “pedagogical approach” to TNA [9]. Whilst this model helps the TNA coordinator select the appropriate approach for the desired outcome, it does not provide any guidance as to how to conduct an assessment that is both comprehensive and effective. As a result, this approach could be a waste of time and resources.

Acknowledging the above challenges and limitations, investigators from the United Kingdom (UK) in 1996 initiated efforts towards a cost-effective and psychometrically sound TNA tool for the healthcare industry. The *Hennessy-Hicks Training Needs Analysis (TNA) Questionnaire,* referred as “TNA” from here on after, was developed to identify individual training needs, organizational requirements, and targeted training strategies [10, 11]. Since its development, the TNA tool has been psychometrically tested and used for a variety of purposes among several settings and populations. It has a proven track record for use with primary healthcare teams, district and practice nurses, nurse practitioners (NPs), and health visitors in the UK [10–13]. The TNA has been shown to minimize response bias and provide reliable information about current performance levels, skill areas most in need of further development, and how to best achieve optimal results. This knowledge facilitates organizations with priority-setting and policy development, as well as with evaluation of their continuous professional development (CPD) programs.

In 2011, the TNA developers licensed the tool to the World Health Organization (WHO) for online use and dissemination through the Workforce Alliance website [14]. With rising calls for evaluating training and competency to regulate nursing practice in the Americas [15], stemming from the *“Global strategy on human resources for health: Workforce 2030”* [16], our motivation for this project was twofold. First, the lead investigator’s experience with translating, adapting, and applying the TNA instrument in another language, as part of an action research PhD dissertation. Second, the team’s affiliation with a WHO Collaborating Center (WHOCC) that promotes global capacity building for nurses and midwives as well as educational quality improvement (QI). Therefore, this integrative review aimed to describe TNA tool utilization across the globe and critically appraise the evidence of its impact in CPD across disciplines and settings.

## Methods

The Hennessy-Hicks TNA Questionnaire and Manual [14] was accessed through the WHO Workforce Alliance website and was carefully reviewed to determine initial intended use. The tool consists of a one-page demographics section, an open-ended question, and a 30-item questionnaire which covers core clinical tasks, arranged into five sub-sections; research/audit, administrative/ technical, communication/teamwork, management/supervisory, and clinical activities. Respondents rate each item on a seven-point scale according to two criteria: “*How critical the task is to the successful performance of the respondent’s job”* and “*How well the respondent is currently performing the task.”* Ratings for criterion A (Criticality Index) provide an overall occupational profile of the job, and those for criterion B (Skill Index) the level of performance. Subtracting the scores (criterion A – criterion B) in each task provides a Training Needs Index. The accompanying manual offers instructions, data analysis, and customization for use in one’s own environment.

To allow for cross-country and cross-setting comparisons, the investigators adopted the Knowledge to Action (KTA) Framework developed by Graham et al. [17]. Knowledge translation is “a dynamic and iterative process that includes the synthesis, dissemination, exchange and ethically sound application of knowledge” [18]. The KTA process conceptualizes the relationship between knowledge creation and action as a “funnel”. Knowledge is increasingly distilled before it is ready for application whereas, the action cycle represents the activities needed for knowledge application and integration [17]. By translating knowledge into action, researchers can effectively address the “know-do” gap in healthcare practice [19].

### Search strategy – eligibility criteria

**Fig. 1:**
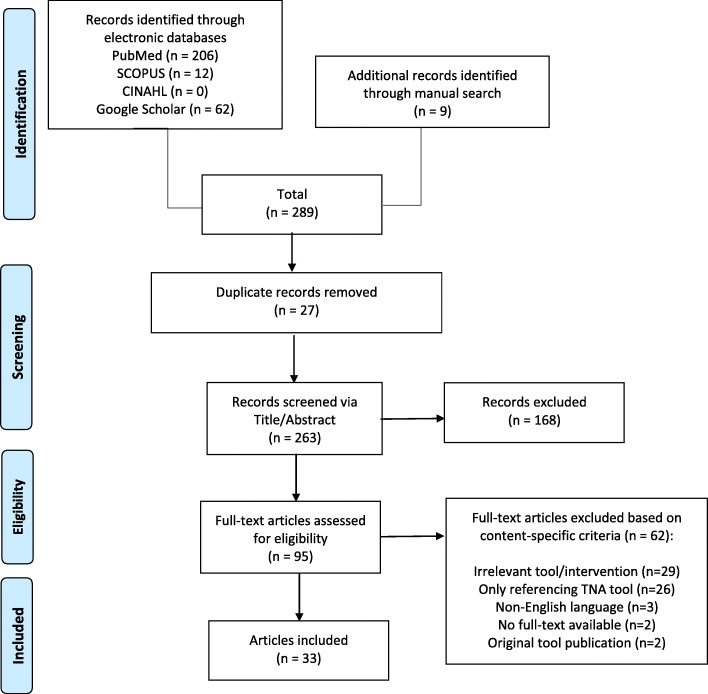
PRISMA Search Strategy for TNA Tool

The Preferred Reporting Items for Systematic Reviews and Meta-Analyses (PRISMA) guidelines were adhered to in the conduct and reporting of this systematic integrative review [20]. An electronic database search in PubMed, Scopus, CINAHL, and Google Scholar was performed using the following strategy: ((“Surveys and Questionnaires”[Mesh] OR tool* OR measure* OR questionnaire* OR survey* OR scale* OR instrument*) AND (Hennessy OR Hicks OR Hennessy-Hicks OR “Hennessy-Hicks Training Needs Assessment Questionnaire”) AND (nurs* OR training-needs)).” All full-text, peer reviewed articles, dissertations or theses published in English language since 1996 were included. Additionally, a targeted manual search of grey literature and listed references was carried out. A total of 289 articles were retrieved and duplicates were removed with the use of Sciwheel Reference Manager. The resulting 265 articles were first screened by title/abstract, and then, 97 full-text articles were assessed for eligibility. During screening and eligibility steps, inclusion was determined if any of the following content-specific criteria were met: a) study using the original TNA tool, b) psychometrics study carrying out translation and/or cultural adaptation of the TNA tool in other languages or countries, and c) study applying or integrating an adapted TNA version. The above search strategy, along with reasons for excluding articles, is depicted in Fig. 1.

### Data extraction and quality appraisal

Initial title and abstract screening was conducted by an independent investigator (SM), with full-text article assessment for eligibility and data extraction carried out by two independent investigators (SM, LT). Any conflicting votes on eligibility of an article were resolved by a third investigator (AM). Appraisal of level of evidence was based on the adapted *Rating System for the Hierarchy of Evidence* [21, 22]. The World Bank Atlas was followed for country classification by income level (high, middle or low) [23]. An inductive thematic analysis of the literature sample was carried out. Interprofessional education (IPE) and collaborative practice were operationalized based on the *Framework for Action on Interprofessional Education and Collaboration*, developed by WHO [24]. Hence, both terms ‘interdisciplinary’ and ‘multidisciplinary’, often used interchangeably, were captured. Given the systematic review methodology of this study, no study approval was required.

## Results

A total of 33 articles, published within the last 25 years (January 1996–May 2020), met our inclusion criteria and are presented in Table 1. A summary of their characteristics and appraised evidence is shown in Table 2. The majority of the articles (87.9%) were original research, 9.1% were dissertations or theses, and 3% posters. Thirty-two (97%) of the studies were descriptive or mixed methods (level VI evidence), and one (3%) was an expert opinion (level VIII). Study populations included nurses (72.7%), physicians (12.1%), other healthcare professionals (30%), and health insurance employees (3%). Settings ranged from primary healthcare (57.6%), to acute care (51.5%), and other organizations (6.1%) or companies (3%). As shown in Table 3, a total of 18 countries were represented, with the majority originating from the UK. More than two thirds (69%) focused on high-income countries (HIC), 28% on middle-income countries (MIC), and only 3% on low-income countries (LIC).

**Table 1:** Characteristics of studies using the Hennessy-Hicks TNA tool (*n* = 33)

Author, year [Ref]/ Country	Publication type	Design/Level of evidence^**a**^	Study population/Setting/Sample size	Used original or adapted or translated TNA tool	Findings/ Implications	TNA theme^**b**^
Adewole et al., 2019 [25] Nigeria	Article (original research)	Cross-sectional survey / Level VI	Other healthcare professionals / healthcare organizations / 234 participants	Used adapted English TNA tool	-Gaps in health insurance industry personnel capacity - Low performance coverage of the social health insurance scheme - Preferred training through courses rather than organizational change -Stakeholders in Nigeria should pay attention to personnel training	Individual
Aw and Drury, 2016 [26] Singapore	Article (original research)	Mixed methods (surveys and focus groups) / Level VI	Nurses / acute care / 91 nurses were surveyed and 19 participated in focus groups	Used adapted English TNA tool	- Training needs for ophthalmic nurses -Develop tailored education programs to meet workforce needs (clinical exposure, patient education)	Team/IP
Barratt and Fulop, 2016 [27] UK	Article (original research)	Descriptive survey / Level VI	Other healthcare professionals / Healthcare organizations / 203 respondents from 20 healthcare and public health organizations	Used adapted English TNA tool	-*Key tasks:* evaluation, teaching, making do with limited resources, coping with change and managing competing demands, assessing relevance of research -*Training priorities:* evaluation; finding, appraising, and applying research evidence; data analysis -*Key barriers:* time and resources, lack of institutional support for research -*Implications:* improved ability of health organizations to use research and participate in knowledge generation can lead to improved healthcare and population health.	Organizational
Carlisle et al., 2012 [8] Australia	Article (original research)	Questionnaire survey / Level VI	Nurses / acute care / 72 nurses from 3 public hospitals	Used original English TNA tool	-Tested the occupational profile construct measurement model and found that it fits the original factorial structure determined by Hicks et al. (1996) - TNA dimensions demonstrated tool’s predictive validity -Organizational factors and demographic variables play a significant role in determining the occupational profile of nurses and their training needs.	Individual
Eyres and Lima, 2018 [28] Australia	Poster	Cross-sectional survey / Level VI	Nurses / acute care / 280 responses from 10 hospital units	Used adapted English TNA tool	-Nurses performed best in communication and teamwork; this was most important for successful job performance -Training need areas: cardiovascular, respiratory, pediatrics, diabetes, communication, and mental health -Survey data used for developing specific training and education	Team/IP
Gaspard and Yang, 2016 [29] Saint Lucia	Article (original research)	Questionnaire survey / Level VI	Nurses, other healthcare professionals / health care organizations / 139 respondents	Used adapted English TNA tool	-Need for continuing professional education was rated as highest priority, followed by research/audit activities -Most respondents required training in communication, management, clinical skills, and research methods -Providing training according to needs is vital in developing countries	Team/IP
Hennessey and Hicks, 1998 [30] UK, USA, Australia	Article (original research)	Questionnaire survey /Level VI	Nurses / primary and secondary health care/ 216 respondents in five groups: health visitor, district nurse, primary care, NP, hospital nurse	Used original English TNA tool	-Similar occupational profiles in all three countries -Results were consistent with the roles and education levels ascribed to the five groups -Identified training needs: patient-centered activities, general work skills, teamwork, and research activities *Implications:* -No universal trends in training needs according to locus of practice -Training requirements are specific to the actual role performed and the organizational environment -Requirements must be assessed on a regular basis before education is commissioned in each country.	Individual
Hennessey et al., 2006a [31] Indonesia	Article (original research)	Questionnaire survey / Level VI	Nurses, other healthcare professionals / primary healthcare, acute care / pilot study included 109 respondents, actual study included 524 nurses and 332 midwives	Used translated and culturally adapted into Bahasa Indonesian TNA version	-Tool was reliable and valid; suited for use with both midwives and nurses -Baseline measurement of the quality of nursing and midwifery across Indonesia. *Implications:* TNA use could ensure that the quality of health service and health professionals is monitored and optimized, which is essential for countries with diminished health status.	Team/IP
Hennessey et al., 2006b [32] Indonesia	Article (original research)	Questionnaire survey / Level VI	Other health care professionals / primary healthcare, acute care / 332 midwives	Used translated and culturally adapted into Bahasa Indonesian TNA version	-Midwives’ roles varied significantly by province, but little difference in the roles of hospital and community midwives -All midwives reported significant training needs for all 40 tasks *Implications: i*mportant regional differences in roles of midwives call for suitability of basic and post-basic education	Individual
Hennessey et al., 2006c [33] Indonesia	Article (original research)	Questionnaire survey / Level VI	Nurses / primary healthcare, acute care / *n* = 524	Used translated and culturally adapted into Bahasa Indonesian TNA version	-Significant differences in job profile across nurses from different provinces -Roles of hospital and community nurses were fairly similar -All nurses reported significant training needs for all 40 tasks -Nurse role is not as diverse as expected, which may reflect the lack of central registration system and quality framework. *Implications:* results provide a rigorous and reliable approach to defining occupational roles and continuing education needs of Indonesian nurses	Individual
Hicks and Hennessey, 1997 (J Adv Nurs) [12] UK	Article (original research)	Questionnaire/ Level VI	Practice nurses/ primary healthcare (regional health authority), acute care / *n* = 420	Used adapted English TNA tool	-Communication and teamwork activities were the most important aspect of a practice nurse’s job -Agreement on how a practice nurse’s role is perceived across the sample -Differences, similarities, and priorities between the practice nurse and NP were identified -The use of a psychometrically valid and reliable instrument may establish the parameters for the definition and education of NPs in the UK	Individual
Hicks & Hennessey, 1997 (J Nurs Manag) [34] UK	Article (original research)	Opinion article / Level VIII	47 nurses and their nurse managers	Used original English TNA tool	−29 nurse/manager pairs showed consensus with respect to training interventions, while the remainder showed no accord. -Overall agreement within each professional sample suggested a common view of training needs *Implications:* a mutually agreed training program that incorporates the organization’s objectives and the individual’s training requirements can be achieved with minimum conflict and an enhanced understanding of both parties’ agendas.	Team/IP
Hicks & Hennessey, 1998 [35] UK	Article (original research)	Questionnaire survey / Level VI	Nurses / acute care / 50 nurses and nurse managers	Used adapted English TNA tool	-Overall high agreement between nurses and managers, regarding NP role definition and the essential training requirements, with somewhat different opinions by medical staff. -Attempt at a first-stage definition of the NP	Team/IP
Hicks & Hennessey, 1999 [13] UK	Article (original research)	Questionnaire survey / Level VI	Nurses / primary healthcare, acute care / 49 acute care nurses and 420 community nurses	Used original English TNA tool	-Both groups perceived advanced clinical activities, including examination and diagnosis, and a range of research activities to be central to NP role. -The primary care sample reported business and management activities as essential skills, while the acute sector nurses regarded communication skills, autonomy and risk management to be more important *Implications:* rather than trying to construct a universal definition of the NP role for all contexts, a generic description, with further specificity added for each of the key contexts of NP service delivery is more realistic.	Individual
Hicks & Hennessey, 1999 [36] UK	Article (original research)	Questionnaire survey / Level VI	Nurses / primary healthcare, acute & tertiary care / 100 participants from 7 NHS trusts	Used adapted English TNA tool	-TNA showed both common training needs and skill deficits relevant to locality and clinical area -Using more objective information allows for commissioning of customized research skills to meet the needs of both the local organization and its employees *Implications:* limited training budgets can be more effectively targeted	Organizational
Hicks & Hennessey, 2001 [37] UK	Article (original research)	Questionnaire survey / Level VI	Practice Nurses enrolled in NP program / 15 nurses participated, 11 completed the course of interest	Used adapted English TNA tool	-Overall, the course reduced skill deficits in all but seven tasks; six related to research and audit and one to clinical examination. -TNA can identify specific training requirements of a professional group to inform educational curricula -TNA can assess the degree to which course provision has met training needs and which skill deficits need to be targeted.	Individual
Hicks & Tyler, 2002 [38] UK	Article (original research)	Questionnaire survey / Level VI	Nurses / primary healthcare/ 388 family planning nurse practitioners (FPNP)	Used adapted English TNA tool	-Role of FPNP is more extended -TNA tool is valid and reliable *Implications:* FPNP role definition can be used to inform educational programs and assess their efficacy	Individual
Hicks & Fide, 2003 [39] UK	Article (original research)	Questionnaire survey / Level VI	Non-specialist breast care nurses / primary healthcare, acute care / *n* = 119	Used adapted English TNA tool	-Significant educational needs were identified across all 30 items −13 out 14 cancer-related items were among the top 19 training needs -Most important tasks: communication/team-work, self-management, awareness, of specific psycho-social problems, knowledge of specific clinical issues, and management of care *Implications:* results should inform continuing educational development for non-specialist nurses caring for breast cancer patients	Individual
Hicks & Thomas, 2005 [40] UK	Article (original research)	Questionnaire survey / Level VI	Nurses, physicians/ primary healthcare/ 54 respondents	Used adapted English TNA tool	-Both groups of community sexual health professionals required training and CPD in a range of areas, including clinical, research, legal, professional, and communication tasks -TNA is a valid instrument -TNA approach allows for optimum use of limited educational budgets by targeting actual training needs of staff and promoting multidisciplinary team working *Implications:* potential for reducing variations in the quality of community sexual health care services	Team/IP
Holloway et al., 2018 [41] New Zealand	Article (original research)	Questionnaire survey / Level VI	Nurses / primary healthcare/ 18 respondents	Used adapted English TNA tool	-TNA identified 10 priority training needs for regional primary healthcare nurses -Preference for organizational development approaches -Usefulness of TNA structured approach to commissioning for continuing education *Implications:* 1) nursing services are expected to respond to changing health care needs, and 2) educational providers should respond with evidence-based curricula that address training needs	Individual
Kilic et al., 2014 [42] Turkey	Article (original research)	Mixed methods / Level VI	Other healthcare professionals/ healthcare organizations / 46 participants	Used adapted English TNA tool	-Considerable research on non-communicable diseases is concentrated in select areas -Great need for training junior researchers, but training alone is not sufficient -Lack of coordination between governmental institutions and researchers *Implications:* prioritization of financial resources can close the gap in areas which do not receive research attention, such as social determinants of health	Individual
Maher et al., 2017 [43] Ireland	Article (original research)	Descriptive survey / Level VI	Physicians / acute care / 547 respondents	Used adapted English TNA tool	-Doctor-patient communication was ranked highest for importance and level of current performance -Workload/time organization and stress management were skills with highest deficiency -Resilience training, management, and communication skills were preferred areas for future continuing professional development (CPD) -All respondents favored interactive, hands-on sessions -Course completion and preference patterns differed significantly across clinical specialties *Implications:* importance of considering individual needs and preferences across clinical specialties to facilitate more CPD programs.	Individual
Maher et al., 2018 [44] Ireland	Article (original research)	Questionnaire survey / Level VI	General practitioners (GPs)/ primary healthcare/ 719 respondents	Used adapted English TNA tool	-GPs identified doctor-patient communication as the most important and best-performed skill -Discrepancies between perceived importance (high) and current performance (low) emerged for time/workload management, practice finance and business skills -GPs preferred primary care and non-clinical topics for future CPD -Flexible CPD methods were important -Gender and practice location significantly influenced CPD participation and course preference	Individual
Markaki et al., 2007 [45] Greece	Article (original research)	Questionnaire psychometrics / Level VI	Nurses, other healthcare professionals / primary healthcare / 10 healthcare professionals in pilot test and 55 in implementation study	Used translated and culturally adapted into Greek TNA version	-Translated and culturally adapted TNA Greek version showed good psychometric properties: 1) satisfactory internal consistency and reproducibility, 2) significant positive correlations between respondents’ current performance levels on each of the research items and research involvement, indicating good validity *Implications:* tool is suitable to assess professional development needs of nursing staff in Greek primary healthcare settings.	Team/IP
Markaki et al., 2009 [46] Greece	Article (original research)	Questionnaire survey / Level VI	Nurses, other health care professionals / primary health care / 119 participants	Used translated and culturally adapted into Greek TNA version	-Significant training needs were reported by all staff, mainly in research/audit and clinical skills -No significant differences between 2-year degree graduates and 3- or 4-year degree graduates (RNs, midwives, and health visitors) *Implications:* regional health authorities should implement a systematic overview of skill deficits in relation to skill requirements to enhance on-the-job training targeting group-specific, local needs	Team/IP
Moty, 2013 [47] USA	Thesis	Questionnaire survey / Level VI	Other / Contract Research Organization / 33 participants in first survey and 26 in second	Used adapted English TNA tool	-Incorporating end users’ input through surveys to optimize portal design led to more positive opinions about portal technology and an increase in desire to use technology in the future *Implications:* utilizing change management methodology could mitigate resistance to change	Organizational
Mwansisya et al., 2020 [48] Tanzania	Article (original research)	Questionnaire survey / Level VI	Nurses, other healthcare professionals / primary healthcare, acute care / 153 participants	Used translated and culturally adapted into Kiswahili TNA tool	-Adapted tool was reliable and valid for identifying training needs in Reproductive, Maternal and Neonatal Health care settings *Implications:* large sample size studies are required to test the use of this translated version in wider health care systems	Team/IP
Ngidi, 2012 [49] South Africa	Dissertation for MBA (School of Business)	Questionnaire survey / Level VI	Medical managers in public hospitals / acute care / 30 respondents in KwaZulu-Natal	Used adapted English TNA tool	-All audited tasks were relevant and overall performance was perceived as high -Training initiatives targeting medical managers should combine informal and formal training methods *Implications:* 1) need for well-trained hospital managers in developing countries, and 2) more qualitative research could provide insight into the context of training needs	Individual
Pavlidis et al., 2020 [50] UK, Greece, Bulgaria, Poland, Italy	Article (original research)	Questionnaire survey / Level VI	Formal and informal caregivers in health and social care (nurses, nursing assistants, home care aides) / primary healthcare / 550 caregivers	Used adapted English TNA tool (UK) – only 10/30 items were used Used translated and culturally adapted TNA tool (Greek, Bulgarian, Polish, and Italian language versions) – only 10/30 items used	-Basic nursing skills, specialization, and training in psychology-related skills emerged as common training needs with some variation by country -Training in advanced health care systems, time management, emotion regulation and communication raise capacity of caregivers employed in health and social care *Implications:* training in basic nursing skills for managing non-communicable diseases (diabetes, stroke, dementia) should be part of CPD programs for European caregivers.	Individual
Shongwe, 2019 [51] South Africa	Article (Research report)	Questionnaire survey / Level VI	Nurses and qualified nursing assistants (QNAs) / acute care / 103 RNs and QNAs from pediatric and neonatal units in 6 hospitals	Used adapted English TNA tool	-TNA identified 15 high level learning needs -Learning needs were skewed towards clinical skills and direct care of neonates and children -There was no statistically significant difference in the overall learning needs between RNs and QNAs -In-service planners and providers should be aware of what nurses in pediatric settings need to learn *Implications:* strengthening of undergraduate curriculum on pediatric nursing and introduction of child nursing specialty programs in the Eswatini nursing education is recommended.	Team/IP
Singh, 2015 [52] India	Article (original research)	Questionnaire survey / Level VI	Nurses, physicians/ Acute care / 274 faculty, senior residents, and nurses	Used adapted English TNA tool	-Studied at the micro level (tasks/job analysis) nurses’ training needs in a tertiary care specialty hospital in Northern India -TNA identified training needs in patient care, research domain, managerial/administrative domain, and communication domain -Nurses identified their needs relevant to their positions and perceived their performance as higher than physicians	Team/IP
Tyler & Hicks, 2001 [53] UK	Article (original research)	Questionnaire survey / Level VI	Nurses / primary healthcare/ 388 family planning nurses	Used adapted English TNA tool	-The nurse prescribing role was defined primarily in terms of prescribing functions, although advanced professional issues, communication, teamwork and business/administration were also mentioned -Research was not identified as being important -The top 15 training needs included 7 research tasks, advanced clinical activities, applied pharmacology, administration and technical activities *Implications:* study offers a role definition of the nurse prescriber in family planning, which can help with curriculum planning and CPD programs.	Individual
Yousif et al., 2018 [54] Sudan	Article (original research)	Questionnaire survey / Level VI	Other healthcare professionals / school / 29 faculty of Dentistry at University of Gezira in Sudan	Used adapted English TNA tool	-TNA showed an urgent need for all competencies except three -Priorities for improvement were ranked as follows: research, leadership, health professional education, managerial, community development, teaching and learning skills. -University-level education and development center should focus on emerged training needs of staff	Team/IP

^a^Level of evidence appraisal based on the adapted *Rating System for the Hierarchy of Evidence* [21, 22]

^b^Themes: Individual, Team/Interprofessional (IP), Organizational

**Table 2:** Summary of characteristics and evidence appraisal (*n* = 33)

**Study population**^**a**^	**n (%)**^**a**^	**Study Setting**^**a**^	**n (%)**^**a**^
Nurses	24 (72.7)	Primary healthcare	19 (57.6)
Other healthcare professionals	10 (30)	Acute care	17 (51.5)
Physicians	4 (12.1)	Health care organization	2 (6.1)
Health insurance employees	1 (3.0)	Health insurance company	1 (3.0)
**Use of TNA tool**	**n (%)**	**Training needs themes**	**n (%)**
Adapted English	23 (69.7)	Individual	17 (51.5)
Translated, or culturally adapted	6 (18.2)	Team / Interprofessional	13 (39.4)
Original English	4 (12.1)	Organizational	3 (9.1)
**Type of publication**	**n (%)**	**Level of Evidence**	**n (%)**
Original research	29 (87.9)	Level VI (descriptive or qualitative or mixed methods study)	32 (97.0)
Dissertation/Thesis	3 (9.1)	Level VIII (expert opinion)	1 (3.0)
Poster	1 (3.0)		

^a^Some articles involved more than one study population or study setting

**Table 3:** Classification of TNA use by country income level and theme

Country classification by income level^**a**^	Individual theme	Team/IP theme	Organizational theme	Total n (%)^**b**^
**High Income Countries**	17	7	3	27 (69.2)
Australia	2	1		3 (7.7)
Greece	1	2		3 (7.7)
Ireland	2			2 (5.1)
Italy	1			1 (2.6)
New Zealand	1			1 (2.6)
Poland	1			1 (2.6)
Singapore		1		1 (2.6)
UK	8	3	2	13 (33.3)
USA	1		1	2 (5.1)
**Middle Income Countries**	6	5		11 (28.2)
Bulgaria	1			1 (2.6)
India		1		1 (2.6)
Indonesia	2	1		3 (7.7)
Nigeria	1			1 (2.6)
Saint Lucia		1		1 (2.6)
South Africa	1	1		2 (5.1)
Tanzania		1		1 (2.6)
Turkey	1			1 (2.6)
**Low Income Countries**		1		1 (2.6)
Sudan		1		1 (2.6)
**TOTAL**^**b**^	**23**	**13**	**3**	**39 (100)**

^a^Income country classification by World Bank [23]

^b^Some articles involved more than one country

In terms of TNA tool use, 4 (12.1%) studies used the original English version instrument, 23 (69.7%) adapted the English version, and 6 (18.2%) translated and culturally adapted the tool. Translation and cultural adaptation of the original TNA was carried out in the following languages/countries: a) Bahasa Indonesian - tested among community nurses and midwives in Indonesia [31–33]; b) Greek - tested among rural primary care nurses, midwives, and health visitors in Greece [45, 46]. In addition, a modified version of TNA was translated and culturally adapted into the following languages/countries: a) Kiswahili – tested among reproductive, maternal, and newborn healthcare workers in Mwanza, Tanzania [48]; b) Bulgarian, Polish, Italian, Albanian, and Romanian – tested among formal and informal caregivers across European Union countries and immigrant communities [50]. Furthermore, a thematic analysis revealed the following three levels in training needs analysis: a) individual level; b) interprofessional or team/unit level; and c) organizational level.

### Individual training needs analysis

As listed in Table 1, a total of 17 studies centered on identifying or targeting needs at the individual level within a specific population of interest across a variety of settings [8, 12, 13, 25, 30, 32, 33, 37–39, 41–44, 49, 50, 53]. Nurses, physicians, midwives, and other healthcare professionals were studied in primary healthcare, acute care, healthcare organizations, and other businesses. All 17 articles applied the TNA tool to determine specific job roles and responsibilities within the targeted setting. Hence, an individual’s perceptions about which tasks were most important for performing their jobs were assessed and captured in column A of the TNA tool. For example, Hicks and Tyler [38] used the tool to determine the required education for family planning nurses in the UK, analyzing the tasks these nurses performed, while also allowing them to indicate their training needs. By comparing the roles of the family planning nurse with the family planning nurse prescriber, the investigators determined the nuances that distinguished the two roles in two consecutive studies [38, 53].

Eight articles determined job profile differences in a variety of geographical locations [12, 13, 30, 32, 33, 38, 50, 53]. Four of these articles compared training needs across different countries or regions; within the UK, USA, and Australia [30], across Indonesia [32, 33], and across five European countries [50]. For example, Pavlidis et al. [50] determined the differences in caregivers’ perceived training needs across the UK, Greece, Bulgaria, Poland, and Italy. Four main training needs were reported as contributing to quality care improvement: a) basic nursing skills; b) specialization in specific conditions (such as diabetes, stroke, dementia); c) training in advanced health care systems; and d) training in psychology-related skills, such as time management, emotion regulation, and communication [50]. Targeting those skills was deemed to improve European caregivers’ capacity in the health and social services sectors.

Existing knowledge gaps, and the most effective training methods to meet individual training needs, were identified across all articles. For example, a study carried out in Ireland, determined medical doctors’ training needs to inform professional development courses [43]. Workload/time organization and stress management were identified as the most pressing needs, while doctor-patient communication was ranked highest for importance and level of current performance. The TNA tool allowed for setting priorities that best met individuals’ training needs, and facilitated managers, local governments to allocate scarce budgets and resources to improve the quality of health care [43]. Similarly, a dissertation study of South African managers in public hospitals revealed that a combination of formal and informal training, enhanced by qualitative research, was the best strategy [49].

### Team/Interprofessional training needs analysis

A total of 13 articles exhibited the team/IP training needs theme, encompassing more than one professional group or focusing on a team or unit as a whole [26, 28, 29, 31, 34, 35, 40, 45, 46, 48, 51, 52, 54]. Target populations included nurses, physicians, and other healthcare professionals across acute and primary care, as well as healthcare institutions. Twelve of the articles were descriptive, qualitative, or mixed methods studies (level VI evidence) whereas, one was an opinion article (level VIII evidence) [34].

Articles under this theme utilized the TNA tool for a wide variety of purposes, including to monitor and optimize quality of care. For instance, Singh and colleagues [52] utilized the TNA tool at a tertiary care hospital in India to evaluate nurses’ training needs. They identified patient care, research capacity, managerial/administrative, and communication as the highest priorities [52]. Other investigators used the tool to determine perception of job roles by nurses and their managers. TNA developers, Hicks and Hennessey [35], used the tool to define the newly established NP role in the UK, providing an operational definition and specific training needs to be targeted. The researchers surveyed all nurses working at advanced clinical levels within an acute sector of the National Health System (NHS) trust. Their triangulation results indicated overall consensus between the nurses and their managers, regarding both the definition of the NP role and the essential training requirements, with somewhat differing opinions by the medical staff [35]. Subsequently, implications in regulating educational provision for NPs in the UK emerged.

The TNA tool was also used to compare training needs and perceptions between different professional groups in the clinical setting, thus allowing for greater analysis of job roles and responsibilities. For instance, Markaki et al. [46] used the tool in Greek primary healthcare centers to determine the occupational profile differences between nurse graduates from 2-year programs and graduates from 3- or 4-year programs. Collected data were then used to determine training gaps to be targeted by future interventions. Determination of training needs at all levels allowed for budgetary analysis and resource allocation for optimum results. Hicks and Hennessy focused on improving the capacity of the NHS trust and its employees by identifying the training needs of practice nurses [34]. In this 1997 study, practice nurses considered communication and teamwork to be the most important aspects of their job, and there was overlap in the training needs identified by the nurses themselves and by their managers. In 2005, Hicks and Thomas [40] analyzed the training needs among professionals delivering community sexual health services and used the data to recommend additional courses within the allocated budget. Similarly, Mwansisya et al. [48] surveyed reproductive, maternal and neonatal healthcare workers within eight districts in Tanzania and provided a baseline of training needs in a low middle-income country. Another study, carried out at a School of Dentistry in a Sudanese university, showed that faculty and staff prioritized academic student supervision, data analysis, and effective presentation skills [54]. The survey also revealed knowledge deficits related to legislation and community engagement. The investigators concluded that effective targeting of these group training needs would require development of university-wide policies for training [54].

### Organizational training needs analysis

Three articles focused on improving outcomes for an organization, such as a healthcare system, hospital, or business [27, 36, 47]. Targeted populations were nurses, physicians, other healthcare professionals, and business employees. Barratt and Fulop [27] applied the TNA tool to improve use of research, and knowledge generation participation, across healthcare and public health organizations in London and Southeast England. In doing so, the investigators identified key tasks, priorities, and barriers to building research capacity, such as assessing the relevance of research and learning about new developments [27]. An earlier study by Hicks and Hennessey explored the issue of evidence-based clinical care within the context of diminishing resources in the British NHS [36]. A TNA survey from seven NHS trusts showed common training needs and skill deficits in relevance to locality and clinical area. The authors concluded that targeting the real skill deficits of the workforce, as well as the personnel most in need of training, was essential for effective integration of evidence-based care within routine practice [36]. Last, Moty [47] focused on technology improvement at a contract research organization to incorporate user feedback into a portal system through the TNA tool. By incorporating end users’ input to optimize portal design, more positive opinions about portal technology could be solicited, and desire to use technology could be increased [47]. All three articles demonstrated the tool’s versatility in addressing organizational training needs at a systems level through an integrated approach.

## Discussion

This integrative review synthesized evidence about TNA tool utilization across the globe, and critically appraised its impact in CPD across various disciplines, settings, and countries. The tool proved to be modifiable for different purposes and contexts, without compromising its high validity and reliability. Its flexible design allowed it to be easily adapted to various populations, settings, and cultures while retaining its psychometric characteristics. Hence, the tool’s value as an international instrument for analyzing training needs in the healthcare and education sectors became evident.

### TNA tool utilization across the globe

Following initial development and testing, the TNA instrument was successfully used in the UK to identify individual training needs and trends related to demographics, with an emphasis on development of the NP role at the early stages [12, 13, 30]. Carlisle and colleagues [8] summarized the latent factor structures that affect the occupational profile construct of the TNA scale and examined them within the Australian context. The investigators confirmed the original five-factor model and suggested that the underlying dimensions relating to the occupational profile were perceived to be important for high performance by nurses in Australia [8]. Nevertheless, the majority of studies reflecting individual training needs struggled with low response rates and self-report bias. Therefore, investigators cautioned about conclusions drawn from data that relied on individuals’ own perceptions of their learning needs. At the team/IP level, perception of training gaps and competences among nurses, midwives, physicians, and public health staff in settings ranging from hospitals to rural health facilities emerged throughout 10 countries. Specifically, in Singapore [26], Australia [28], Saint Lucia [29], Indonesia [31], UK [34], Greece [45, 46], Tanzania [48], South Africa [51], India [52], and Sudan [54]. These studies used the TNA tool for primary data collection, with one translation and validation into Bahasa Indonesian language [31] and another into Greek [45]. Training needs of a group of interest were compared to those of other professional groups or team members to tailor CE offerings and optimize IP operations. At the organizational level, UK healthcare institutions and NHS trusts [27, 36] as well as a US contract research organization [47] used the TNA instrument to improve research capacity/utilization, identify key barriers, and mitigate resistance to change. By establishing the relationship between organizational factors (hospitals) and demographic variables, individual occupational competency profiles, as well as team professional development, can be planned and executed by HR departments.

**Fig. 2:**
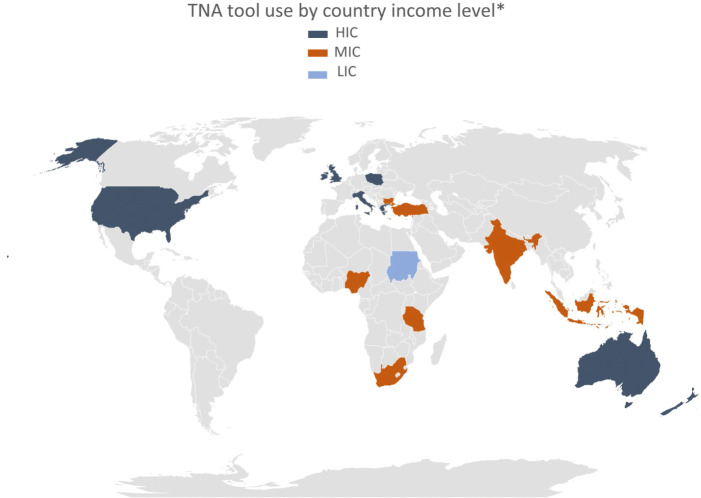
Literature heat map – TNA tool use by country income level. *Income country classification by World Bank [23]

Further synthesis of sampled participating countries, depicted in Fig. 2, revealed that two-thirds of the studies occurred in HICs and one-third in MICs, with only one study stemming from an LIC. Out of 11 studies that used translated TNA versions, three were from Indonesia (MIC), two from Greece (HIC), and one study involved six European countries (4 HICs, 2 MICs). Cross-country comparison by income classification and TNA theme allowed for examination of challenges or limitations in usage of the original, adapted, or translated TNA tool version, and how these were addressed by the investigators. According to Gaspard and Yang [29], how a healthcare professional determines which tasks are essential, and how they perceive their actual performance of that task, may be influenced by several factors. For instance, motivation for continuous learning, a special interest in a particular task, a specific education deficit, and satisfaction or not with unit management. This limitation is addressed by allowing for two ranking systems - where employers also rank employees - to cross-check motivation and establish the need. For example, to determine NP training that would satisfy NHS trust aims, a full training needs analysis of nurses and their immediate supervisors was carried out [34]. The nurses completed the analysis with their own perceptions of training needs, while their managers completed it on behalf of the identified nurse. The resulting mutually agreed training program enhanced understanding of both parties’ agendas and could be achieved with minimum conflict [8, 33, 34, 46]. For Greek nurses in rural PHC settings, appropriate training activities, along with organizational changes, had potentially equal impact on short-term staff development and long-term strategic planning programs [46].

Reported flaws or limitations of the original or adapted/translated TNA tool included: a) small-scale study [8, 26, 37, 40, 42, 45, 46]; b) polling only one organization, or specific unit [26, 29, 37, 42, 49]; c) not surveying employers or stakeholders [28, 29, 48, 51]; d) low participation or item completion rates [34, 38, 45, 46, 53]; e) lack of consensus at a national level regarding training content [45]; and f) focus on individual and team assessment, rather than organizational [25, 39]. Last, one study included only 10 out of the 30 original TNA items, with the rest of items being newly introduced [50], which exceeds the developers’ threshold for psychometrics modification [14]. Furthermore, some investigators recommended further studies to explore TNA’s: a) applicability in a wider healthcare system, b) feasibility as a large-scale survey instrument in secondary and tertiary care settings, and c) usability for collaborative activities, especially through global information technology network teaching programs [34, 45, 48].

The main advantage of the TNA instrument is the accompanying detailed instruction manual, made available by the developers through the WHO Workforce Alliance website [14]. According to this manual, the standard 30-item questionnaire can be tailored to a particular study focus. Up to 8 of the original items can be changed or omitted, and up to 10 new items can be added. To this direction, the developers have included an example of how to adjust the questionnaire ([14], p.21-25). The additional items are to be devised according to an accepted psychometric process for developing questionnaires. For example, a literature review, focus group and interview with relevant personnel should be conducted, with the information distilled into themes, using an approved data-reduction method, such as the Thematic Network Analysis [55]. This provides the core areas from which tailored items are to be constructed. Coverage of the themes (and subsequent items) should be comprehensive and appropriate. These themes will form the basis for new items, which should be in a format similar to that of the standard questionnaire. Modified questionnaires should be piloted with a small sample. The manual includes additional item banks that have been used in other studies, grouped as follows: *“Extended nursing role”*, *“Nurse prescribing”*, *“Specialist care”, “Child abuse / child protection”,* and *“Management”* ([14], p.50-55). This item bank allows for easy tailoring to an investigator’s aim and unique context.

### Global impact in continuous professional development

Regardless of country and profession surveyed, the tool consistently revealed the perceived and assumed training needs, clarified roles, and facilitated CPD at the individual or team level. Sampled literature revealed that there were no universal trends in training needs according to locus of practice, and that training requirements were specific to the actual role performed within an organizational environment. For example, Hicks & Tyler [38] and Hicks & Fide [39] demonstrated that a more targeted, less costly, training program would be optimum, upon surveying the roles and training needs of family planning nurses and breast care nurses in the UK. Organizational factors were shown to determine the occupational profile and training needs of nurses in primary and secondary care across Australia, UK, and the USA [8, 30, 36]. Similarly, most surveyed caregivers in five European countries declared a need for training in the psychosocial aspects of caregiving [50]. Yet, when dealing with stress, caregivers from Italy and Greece had lower needs than those from Poland, UK, and Turkey. Despite the socio-economic differences among countries, all participants faced increasing demands from caregiver burden [50]. For employees in the Nigerian health insurance industry, addressing the gaps through an on-the-job training course was deemed to be the optimum approach [25]. For each country seeking to minimize economic impact and streamline processes, the TNA tool provided an affordable, standardized approach to prioritize and implement an effective CPD program.

Several other studies concurred on the urgent need for flexible and tailored CPD, as an outcome of a comprehensive TNA analysis [26, 33, 40, 44, 46, 52–54]. Especially in middle-income and low-income countries, such as Saint Lucia, South Africa and Sudan, strengthening university-level education for nurses and other healthcare professionals was a key recommendation for evidence-based decision making [29, 51, 54]. In the case of Indonesia, the introduction of TNA in a series of studies, carried out by Hennessy and colleagues [31–33], motivated several junior nurse researchers to pursue and study CPD, as reflected in multiple references of the instrument. Given the prevailing budget constraints and limited accessibility to research funds, it was not surprising that training needs in research competences emerged as top priorities across studies conducted in MICs and LICs. In Turkey, building research capacity among public health professionals to tackle prevailing non-communicable diseases, was seen as a national priority [42]. Following identification of several individual, team and organizational barriers, a comprehensive CPD plan for junior researchers, and a QI plan for governmental institutions were recommended. This latter plan provides a roadmap for addressing the lack of coordination between institutions and researchers, establishing research monitoring and evaluation, and strengthening routine health information systems. Similarly, in Sudan, the top QI priority for university faculty of Dentistry was research, followed by leadership, health professions management, community engagement, and teaching skills [54]. As demonstrated in a UK study, raising the ability of NHS organizations to use research and generate knowledge was tied to improved services and population health [27]. The above findings support the argument for research capacity as an essential component of PHC nursing [56]. Moreover, findings show the critical need for operational integration of standardized QI processes across units or institutions in order to close the gap between theory and practice [57]. Hence, implementation of a regionally based CPD and QI program, stemming from TNA application, could be the solution to a healthcare system in need of reform.

### Strengths and limitations

Integrative reviews allow for the combination of diverse methodologies (i.e., experimental and non-experimental research) in order to more fully understand a phenomenon of concern [58]. By combining data from the theoretical as well as empirical literature, this review could potentially impact evidence-based practice for nursing and other healthcare professions. The ongoing worldwide interest in the Hennessy-Hicks TNA instrument and manual has been a strong incentive for this review. Following personal communication with the developers, they confirmed receipt of many email enquiries, estimated around 100, from around the world asking for permission or clarification about using the tool and informing about outcomes. Unfortunately, there is no cumulative record of requests during the past 25 years. This lack of usage data and repository was further compounded by inability to obtain webpage metrics (i.e. ‘hits’ and ‘downloads’) due to the hosting Global Health Workforce Alliance (GHWA) webpage no longer being maintained [14]. Therefore, no information was available on who has been using the tool nor who is resourcing the archived tool on the GHWA website.

This review was based on an extensive search of four major electronic databases and a targeted manual search of grey literature and cross-listed references. The selected databases (PubMed, CINAHL, Scopus, and Google Scholar) capture a variety of international journals in the nursing, social sciences, and biomedical literature, with relevant hand-searched theses and dissertations also included. All articles were independently reviewed and appraised, using an adapted hierarchy scale for level of evidence [26, 27]. The main limitation stems from the exclusion of any relevant literature published in languages other than English. According to the developers, TNA has been translated into many other languages, including Arabic and Chinese, but there was scarce evidence published in the English literature. Upon appraisal, variation in criteria application, along with cross-country cultural and linguistic variations are also acknowledged. Last, World Bank rankings [23] were based on the 2020 index rather than the year when the study was conducted. Because of the relatively small sample size (*n* = 33), the investigators combined the “low-middle income countries” with the “upper-middle income countries” categories under “middle-income countries” (MIC).

### Implications

The TNA instrument allows for triangulation of a) assessment (identifying and triaging needs); b) needs (gap between what exists and what is required); and c) training (acquiring knowledge, skills or change attitude). Our literature review synthesized the reported use and value of the Hennessey-Hicks TNA tool across settings, populations, and countries, identified the enablers and barriers to its use, and distilled best practice CPD recommendations. Viewed from the KTA Framework perspective, our findings explain how the scholarship of discovery (TNA tool development, psychometrics of adapted or translated versions) leads to the scholarship of integration (translation and cultural adaptation of TNA), and ultimately to scholarship of application (using different versions of the tool across various settings, populations, or countries). As knowledge moves through each stage, it becomes more synthesized and therefore, useful to end-users. Hence, for healthcare professionals, CE should be based on the best available knowledge, the use of effective educational strategies, and the planned action theories to understand and influence change in practice settings [17]. A recent metasynthesis of CPD literature highlights nurses’ belief in CPD, as fundamental to professionalism and lifelong learning, and its importance in improving patient care standards [59]. Yet, it shows a disconnect between nurses’ CPD needs and expectations with the organizations’ approaches to professional development. The authors conclude that access to CPD should be made more attainable, realistic and relevant [59]. By translating TNA evidence into action, health policy makers, administrators, and educators can effectively design appropriate, cost-effective CPD programs with clear priorities to achieve the desirable knowledge, skills and practice, tailored to local needs.

There is also a high level of fit between the adopted KTA Framework and the affiliated WHOCC’s Terms of Reference. Completion of this review coincided with the start of a project by the Registered Nurses Association of Ontario, aiming to develop a repository of measurement tools that can be mapped to the KTA Framework, and to report on their pragmatic and psychometric properties. Given that the TNA measurement tool has been identified as mappable to the KTA framework, the potential synergy between the two groups is promising. Moreover, our findings are aligned with WHO recommendations based on the *“Framework for Action on Interprofessional Education and Collaborative Practice”* [24]. These call for health policy makers to systematically address training needs of the healthcare workforce in order to strengthen IPE and collaborative practice. As tasked by the Pan American Health Organization (PAHO), the affiliated WHOCC aims to enhance the use and dissemination of knowledge resources that build capacity and leadership for nurse and midwife educators. Hence, lessons learned will be used to promote TNA tool application and integration for individual, team/IPE, and organizational improvement across the PAHO region (North/Central/South America and the Caribbean). These steps are both timely and relevant for evaluating training and competency, and for regulating nursing practice in the Americas [15] during the post-pandemic era.

## Conclusion

Since its development in 1996, the TNA instrument has been widely used as a clinical practice and educational quality improvement tool across continents. Translation, cultural adaptation, and psychometric testing within a variety of settings, populations, and countries consistently reveal training gaps along the individual, team/interprofessional, and organizational themes. It is not only applied to identify training needs and demographic trends, but also to prioritize targeted training strategies and CPD programs. Furthermore, it facilitates triaging and allocating limited educational resources, especially in low and middle-income countries. These findings underscore the tool’s effectiveness in addressing the “know-do” gap in global human resources for health by translating knowledge into action.

## Data Availability

All data generated or analyzed during this study are included in this published article.

## Abbreviations

CE: Continuing Education
CPD: Continuous Professional Development
HIC: High Income Countries
IP: Interprofessional
IPE: Inter-Professional Education
KTA: Knowledge to Action
LIC: Low Income Countries
MIC: Middle Income Countries
NHS: National Health System
NP: Nurse Practitioner
PHC: Primary Health Care
QI: Quality Improvement
PAHO: Pan American Health Organization
TNA: Training Needs Analysis
WHO: World Health Organization
